# Supplementary material for: Subtype of Neuroblastoma Cells with High KIT Expression Are Dependent on KIT and Its Knockdown Induces Compensatory Activation of Pro-Survival Signaling
Source: Int J Mol Sci. 2022 Jul 13;23(14):7724. doi: 10.3390/ijms23147724 (PMC9324519; doi:10.3390/ijms23147724)
Supplement: Supplementary file 1 [file ijms-23-07724-s001.zip › Supplementary Material Figures.pdf]

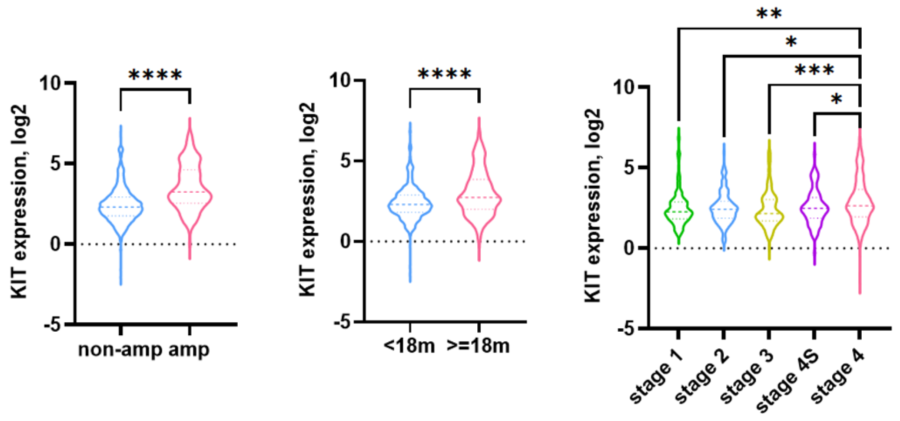

Figure S1. KIT expression in NB tumors with MYCN amplification, different age (>18 or <18 months) and different stages. *- $p < 0.05$, **- $p < 0.01$, ***- $p < 0.001$, ****- $p < 0.0001$ as determined by non-parametric Mann-Whitney test for pair comparisons and by non-parametric ANOVA for multiple comparisons.

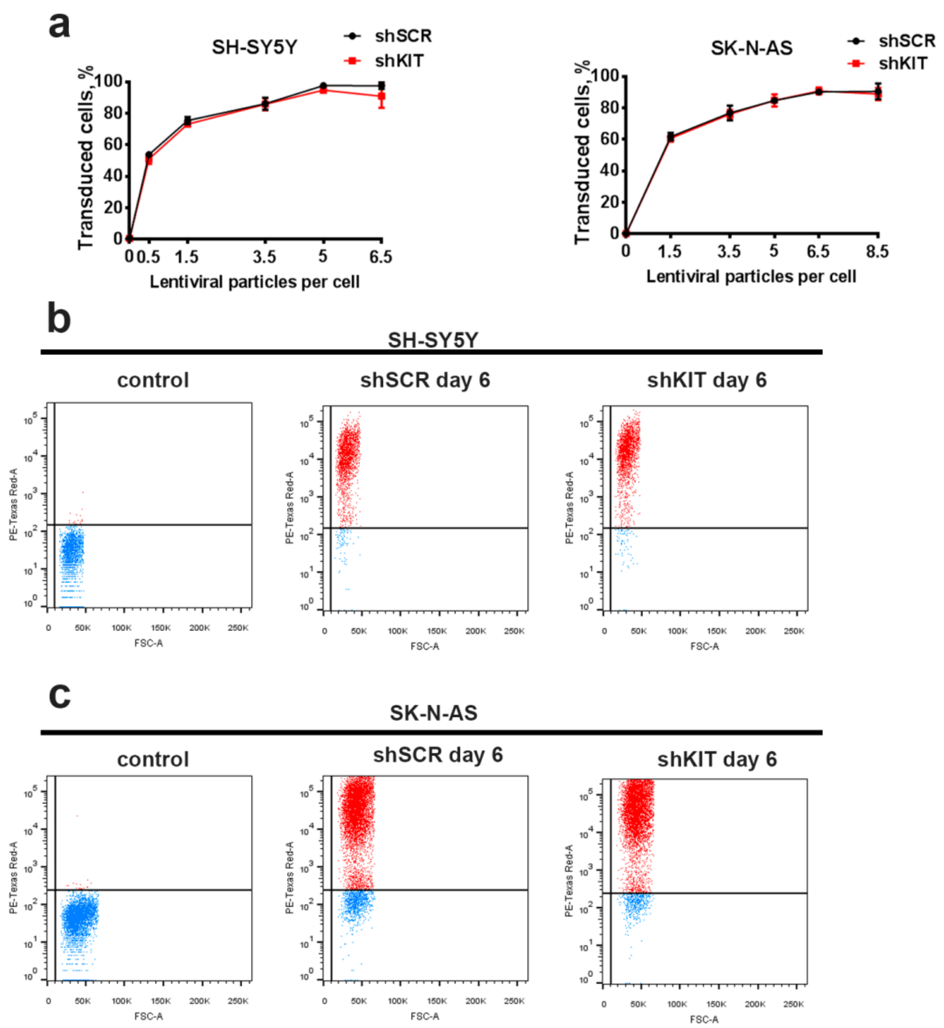

Figure S2. Lentiviral titration and transduction of neuroblastoma cells. a. Lentiviral particle containing supernatant was titrated on both cell lines to determine optimal (at least 90% transduction) amount of lentiviral particles per cell for each cell line and each shRNA vector.

Transduction rates were measured by flow cytometry 72h after transduction. Transduction stability was checked by flow cytometry at day 6 after transduction of SH-SY5Y cells (**b.**) and for SK-N-AS cells (**c.**).

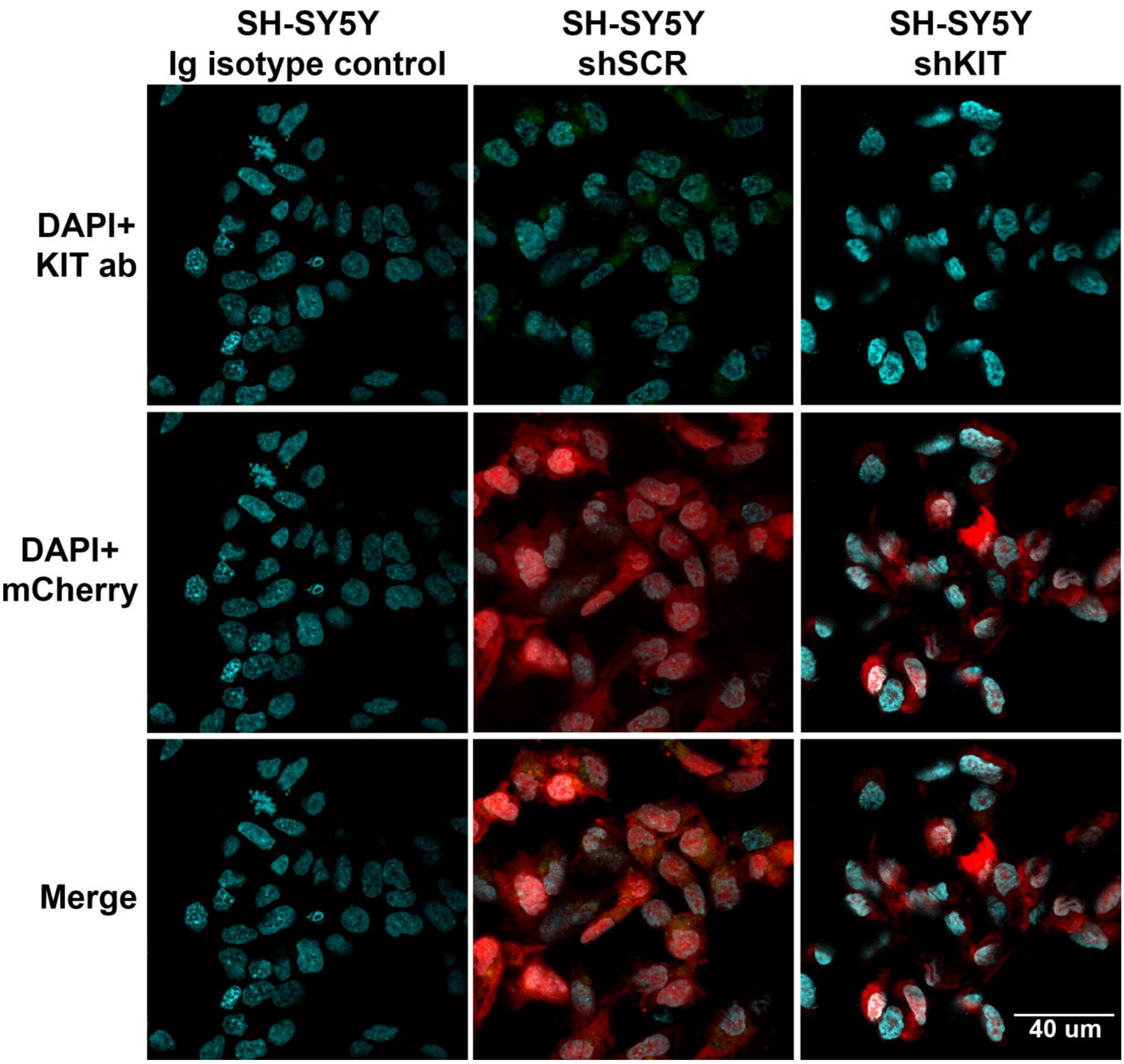

Figure S3. Immunocytochemistry analysis for KIT protein in SH-SY5Y cells 3 days after transduction with shSCR or shKIT lentiviral vectors. Nuclei were stained with DAPI.

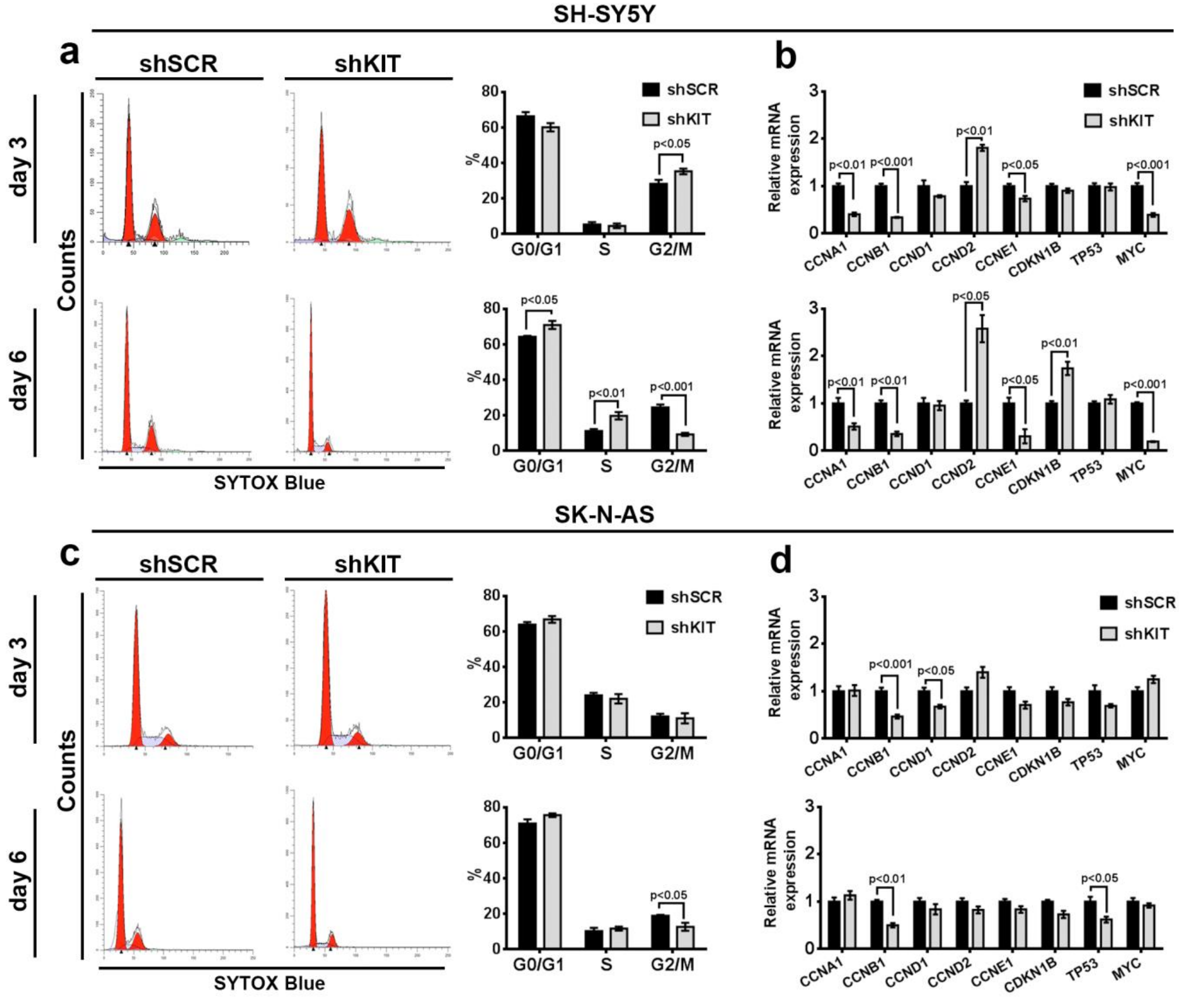

Figure S4. Cell cycle changes after *KIT* knockdown. (a) Distribution of transduced SH-SY5Y cells in different cell cycle phases measured by SYTOX Blue staining on flow cytometer on days 3 and 6 after transduction. Quantitative data is shown on histograms (central panels) as percentage of cells in each phase of cell cycle for three repeats. **(b)** Relative mRNA expression of cyclin genes, p53 and MYC in transduced SH-SY5Y cells measured by real-time RT-PCR on days 3 and 6 after transduction. (**c)** Distribution of transduced SK-N-AS cells in different cell cycle phases measured by SYTOX Blue staining on flow cytometer on days 3 and 6 after transduction. Quantitative data shown on histograms (central panels) as percentage of cells in each phase of cell cycle for three repeats. **(d)** Relative mRNA expression of cyclin genes, p53 and MYC in transduced SK-N-AS cells measured by real-time RT-PCR on days 3 and 6 after transduction.

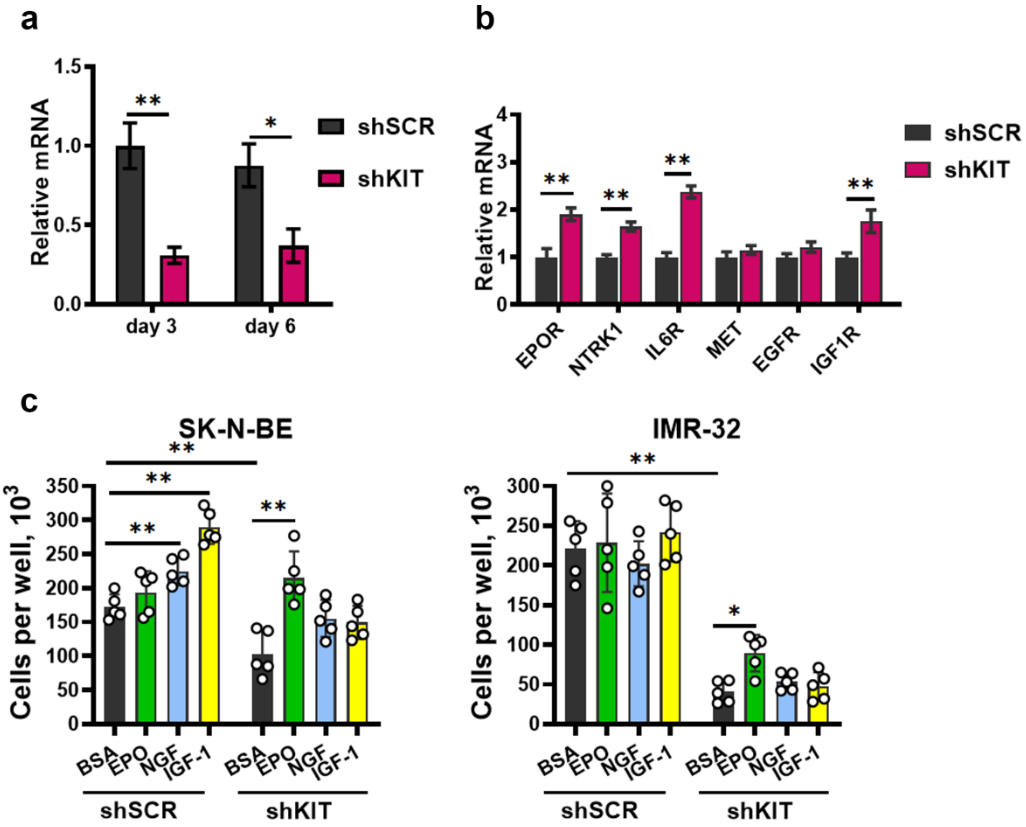

Figure S5. (a). KIT knockdown in SK-N-BE cells after transduction with shRNA lentiviral vectors. **(b)** Changes in expression of growth factor receptors measured by real-time PCR 6 days after KIT knockdown in SK-N-BE cells. **(c)** Effect of KIT knockdown on SK-N-BE and IMR-32 cell proliferation in the presence of 100 ng/ml EPO, IGF-1, or NGF. BSA was used as a mock-treatment control. *- $p < 0.05$, **- $p < 0.01$, as determined by non-parametric Mann-Whitney test.

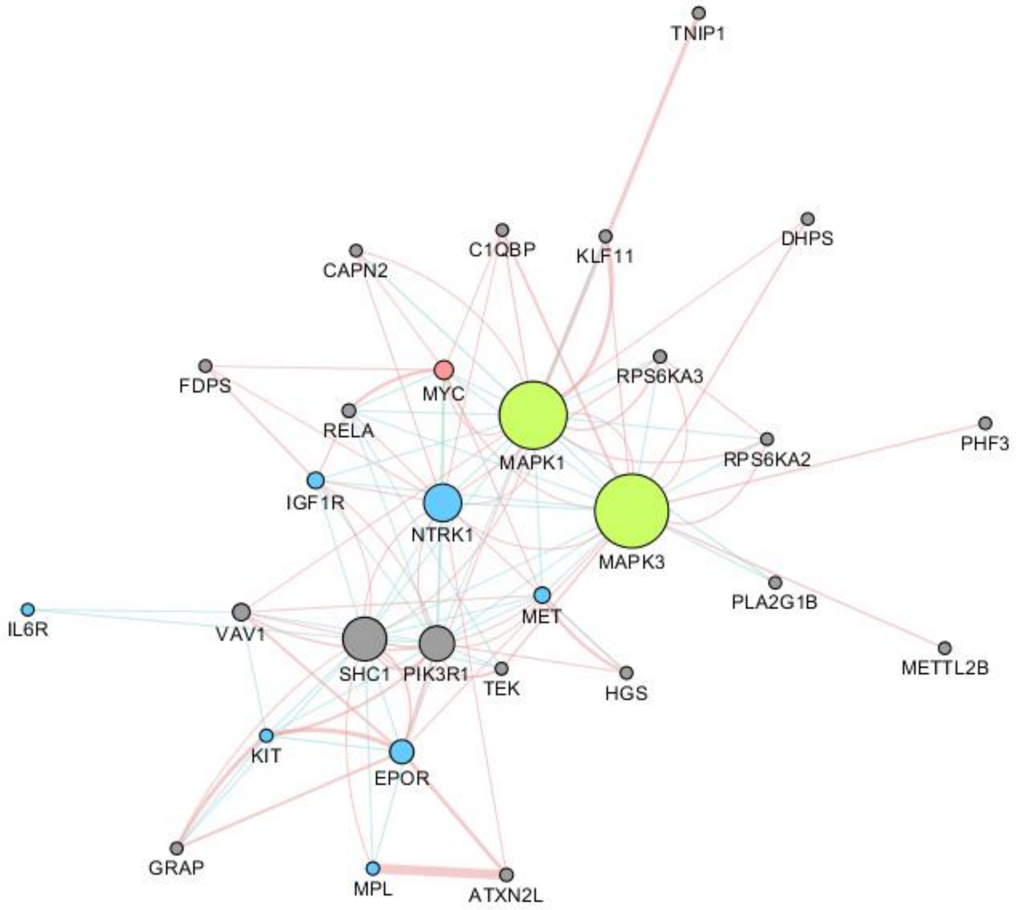

Figure S6. GeneMania generated network.

Table S1. Datasets used for pan-cancer analysis of KIT expression in tumors and associations with immune signature.

Table S2. Pearson correlation between KIT expression and immune signatures in tumors.

Table S3. Pearson correlation between KIT expression and signaling pathway activation in 60 NB tumors.

Table S4. Signaling pathway activity in SH-SY5Y and SK-N-AS cells three and six days after cell transduction with KIT shRNA.

Table S5. Primer sequences used for quantitative real-time PCR.
